# Supplementary material for: Analysis of Oral and Gut Microbiome Composition and Its Impact in Patients with Oral Squamous Cell Carcinoma
Source: Int J Mol Sci. 2024 May 31;25(11):6077. doi: 10.3390/ijms25116077 (PMC11172797; doi:10.3390/ijms25116077)
Supplement: Supplementary file 1 [file ijms-25-06077-s001.zip › ijms-3002469-supplementary.pdf]

Table S1. Clinicopathological characteristics of patients with oral cancer

Category		n = 72
Sex	(male/female)	42/30
Age	average (range)	66.5 (32–92)
T	1	5
	2	35
	3	8
	4	24
N	negative	40
	positive	32
Stage	I	5
	II	29
	III	9
	IV	29

(A)

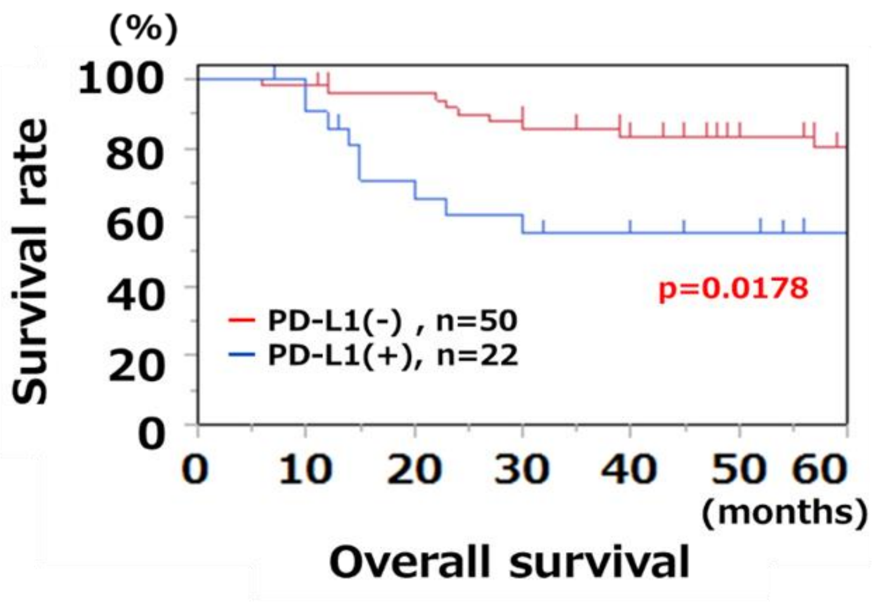

(B)

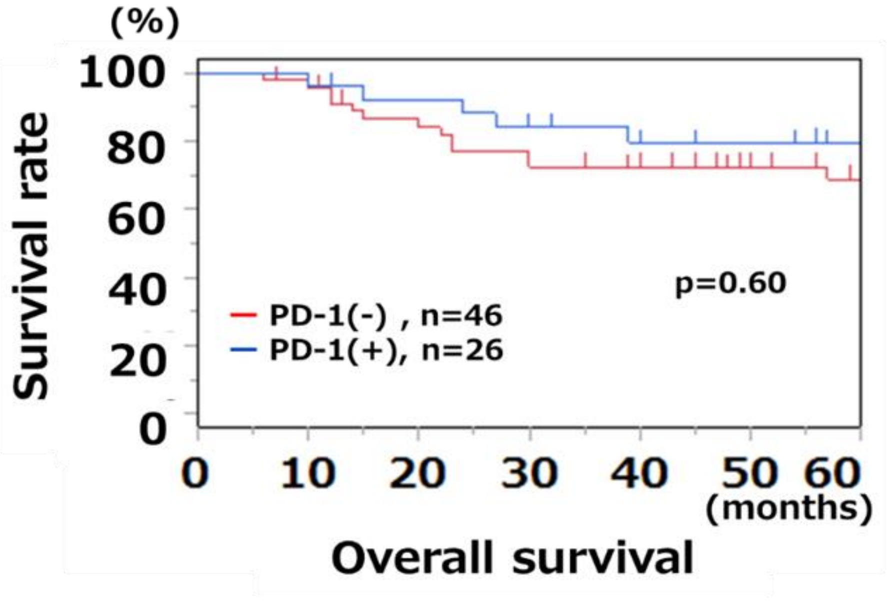

Figure S1. Kaplan–Meier survival curves of patients with OSCC with and without PD-1 or PD-1 expression

Kaplan–Meier curves of overall survival according to PD-L1 (A) and PD-1 (B) expression in OSCC tissues. Each graph indicates the overall survival of patients with positive (blue line) and negative (red line) groups of PD-L1 (A) and PD-1 (B). The statistical differences were determined using the log-rank test. $p < 0.05$, statistically significant difference (n = 72)

OSCC, oral squamous cell carcinoma; PD-1: programmed cell death 1; PD-L1: programmed cell death 1-ligand 1

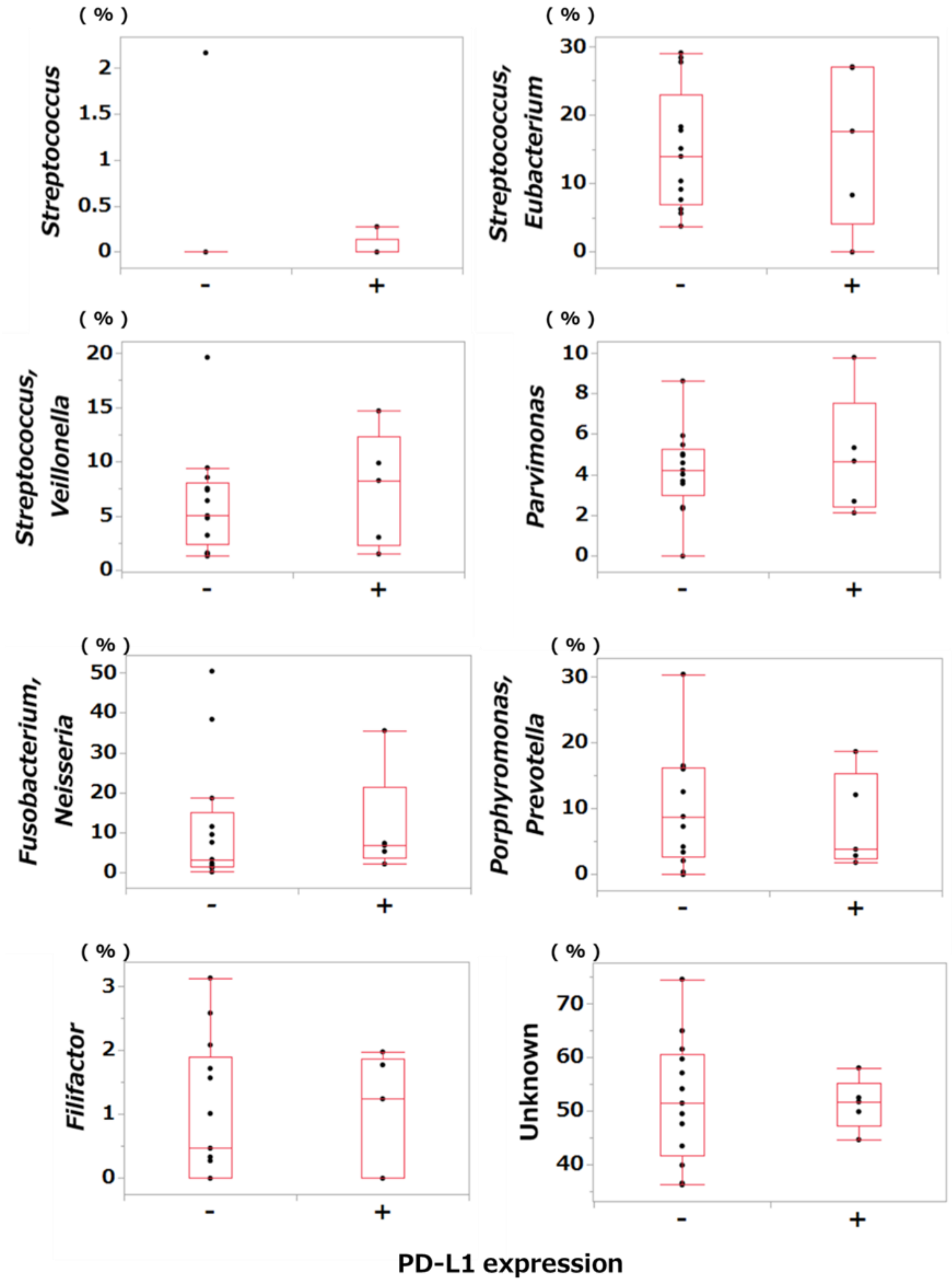

Figure S2. Comparison of the occupancy rate between the PD-L1-positive and PD-L1-negative groups in patients with OSCC. *Hha* I (n = 18: Number of patients with oral cancer who participated in this case-control study)

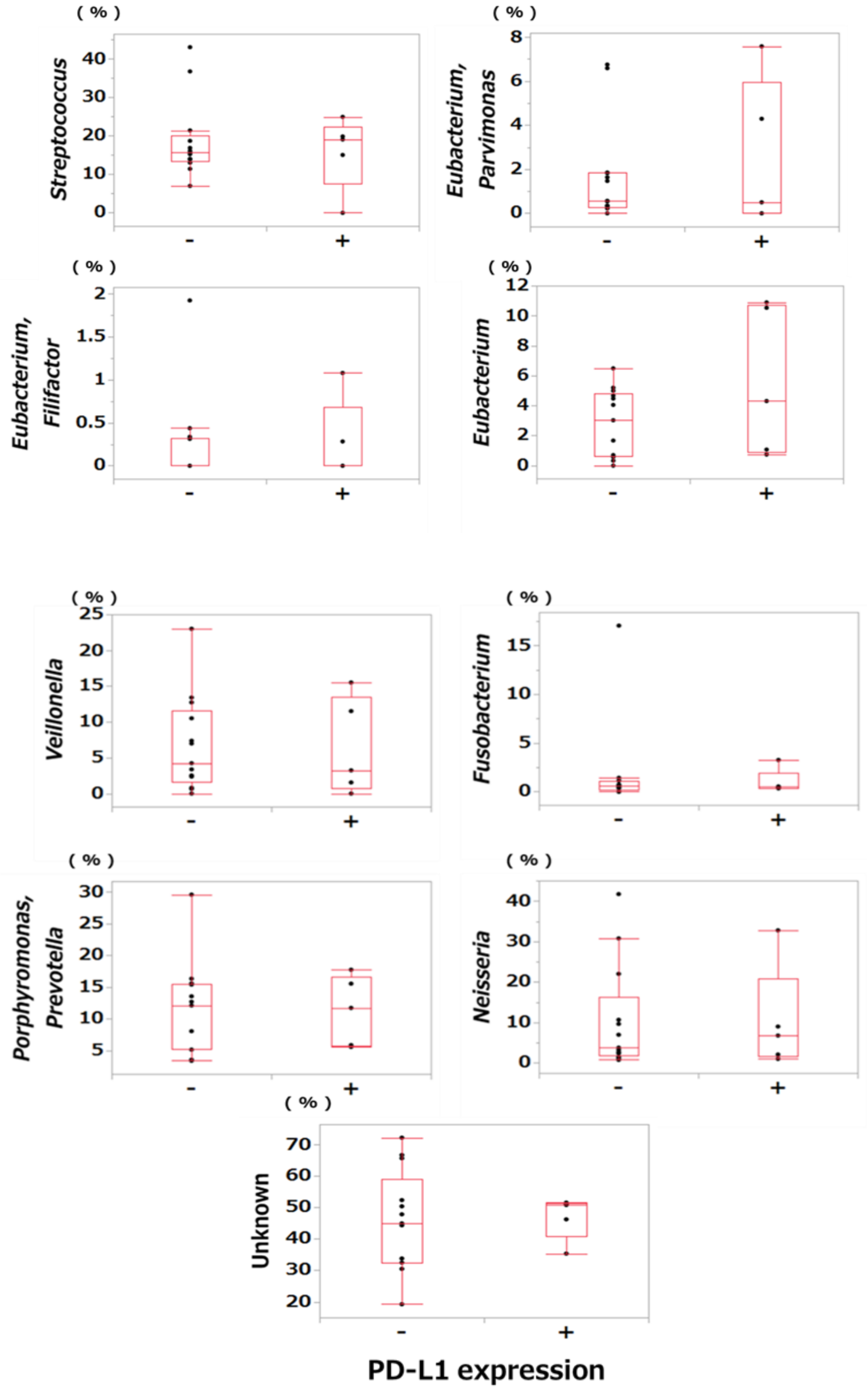

Figure S3. Comparison of the occupancy rate between the PD-L1-positive and PD-L1-negative groups in patients with OSCC. *Msp* I (n = 18: Number of patients with oral cancer who participated in this case-control study)
